# [1,2-Bis(di­cyclo­hexyl­phosphan­yl)-1,2-dicarba-*closo*-dodeca­borane-2κ^2^ *P*,*P*′]di-μ-chlorido-1:2κ^4^ *Cl*:*Cl*-di­chlorido-1κ^2^ *Cl*-dimercury(II)

**DOI:** 10.1107/S1600536814006096

**Published:** 2014-03-22

**Authors:** Liguo Yang

**Affiliations:** a Department of Chemistry, University of Science and Technology Beijing, Beijing 100083, People’s Republic of China

## Abstract

The title compound, [Hg_2_Cl_4_(C_26_H_54_B_10_P_2_)], was synthesized by the reaction of 1,2-bis­(di­cyclo­hexyl­phosphan­yl)-1,2-dicarba-*closo*-dodeca­borane with HgCl_2_. Both Hg^II^ atoms show a distorted tetra­hedral coordination geometry, provided by the two bridging chloride anions and the P atoms of the diphosphanyl ligand for one metal atom, and by two bridging and two terminal chloride anions for the other. The five-membered HgP_2_C_2_ chelate ring assumes an envelope conformation, with the Hg^II^ atom displaced by 0.1650 (5) Å from the mean plane of the other four atoms (r.m.s. deviation = 0.002 Å). In the crystal, B—H⋯Cl interactions link the molecules, forming a supramolecular chain along the *a*-axis direction.

## Related literature   

For related structures, see: Su *et al.* (2008 ▶); Yang *et al.* (2011 ▶); Zhang *et al.* (2006 ▶). For the synthesis and structure of 1,2-bis­(di­cyclo­hexyl­phosphan­yl)-1,2-dicarba-*closo*-dodeca­borane, see: Su *et al.* (2007 ▶).
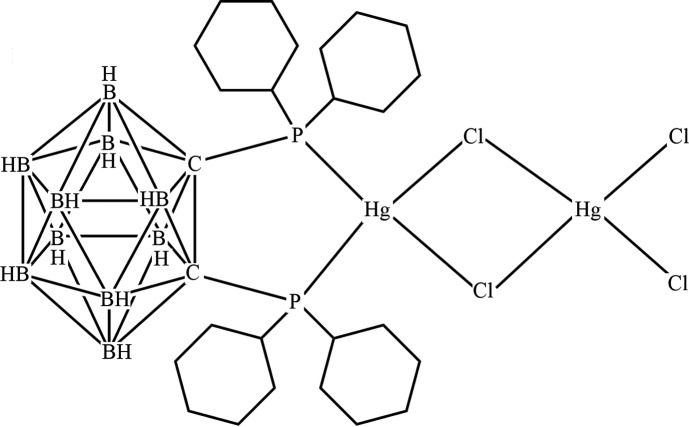


## Experimental   

### 

#### Crystal data  

[Hg_2_Cl_4_(C_26_H_54_B_10_P_2_)]
*M* *_r_* = 1079.71
Monoclinic, 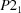

*a* = 10.2233 (11) Å
*b* = 16.6581 (18) Å
*c* = 11.7146 (15) Å
β = 95.000 (1)°
*V* = 1987.4 (4) Å^3^
*Z* = 2
Mo *K*α radiation
μ = 8.08 mm^−1^
*T* = 298 K
0.31 × 0.28 × 0.26 mm

#### Data collection  

Bruker SMART1000 CCD diffractometer
Absorption correction: multi-scan (*SADABS*; Sheldrick, 1996 ▶) *T*_min_ = 0.188, *T*_max_ = 0.228
10374 measured reflections
6455 independent reflections
5998 reflections with *I* > 2σ(*I*)
*R*_int_ = 0.047

#### Refinement  

*R*[*F* ^2^ > 2σ(*F* ^2^)] = 0.049
*wR*(*F* ^2^) = 0.148
*S* = 1.08
6455 reflections
397 parameters
61 restraints
H-atom parameters constrained
Δρ_max_ = 1.33 e Å^−3^
Δρ_min_ = −1.45 e Å^−3^
Absolute structure: Flack (1983 ▶), 2819 Friedel pairs
Absolute structure parameter: −0.010 (11)

### 

Data collection: *SMART* (Siemens, 1996 ▶); cell refinement: *SAINT* (Siemens, 1996 ▶); data reduction: *SAINT*; program(s) used to solve structure: *SHELXS97* (Sheldrick, 2008 ▶); program(s) used to refine structure: *SHELXL97* (Sheldrick, 2008 ▶); molecular graphics: *SHELXTL* (Sheldrick, 2008 ▶); software used to prepare material for publication: *SHELXTL*.

## Supplementary Material

Crystal structure: contains datablock(s) I, global. DOI: 10.1107/S1600536814006096/rz5109sup1.cif

Structure factors: contains datablock(s) I. DOI: 10.1107/S1600536814006096/rz5109Isup2.hkl

CCDC reference: 992563

Additional supporting information:  crystallographic information; 3D view; checkCIF report

## Figures and Tables

**Table 1:** Hydrogen-bond geometry (Å, °)

*D*—H⋯*A*	*D*—H	H⋯*A*	*D*⋯*A*	*D*—H⋯*A*
B1—H1⋯Cl3^i^	1.10	2.71	3.791 (16)	169

Symmetry code: (i) 
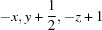
.

**Fig. 1.**
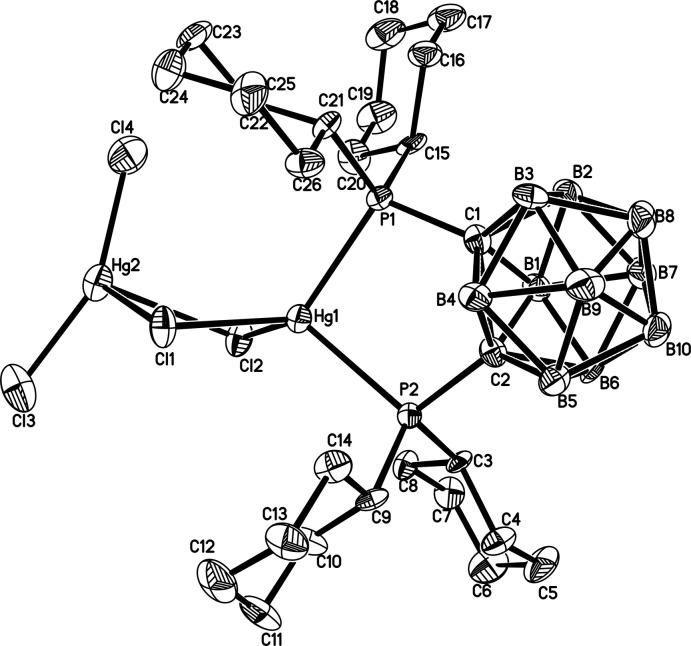
The molecular structure of the title compound with 30% probability displacement ellipsoids for non-H atoms. H atoms are omitted for clarity.

## supplementary crystallographic information

### 1. Comment

The synthesis and structure of 1,2-(PCy_2_)-1,2-C_2_B_10_H_10_ was reported by Su *et al.* (2007). Since then, only a few complexes of this ligand with Ag(I) have been described (Yang *et al.*, 2011). Here we report the structure of this ligand combined with Hg and chloride ions.

As shown in Fig. 1, the coordination of the Hg1 atom is distorted tetrahedral, formed by two Cl^-^ anions and the P atoms of dicyclohexylphosphanyl-*closo*-carborane ligand. The coordination of the Hg2 atom is also distorted tetrahedral, formed by four Cl^-^ anions. The two P—Hg bond lengths are slightly longer than the corresponding bond lengths in the complex [Ag_2_Cl_2_(C_26_H_30_B_10_P_2_)_2_].2CH_2_Cl_2_ (2.5052 (14) Å; Zhang *et al.*, 2006). The P—Hg—P angle is slightly larger than the corresponding value of 89.80 (5) Å for the complex [Ag_2_Cl_2_(C_26_H_30_B_10_P_2_)_2_].2CH_2_Cl_2_ (Zhang *et al.*, 2006). The five-memebered chelate ring is formed by the mercury atom, two phosphorus atoms and two carbon atoms of the carborane skeleton. The torsion angle P1—C1—C2—P2 is -0.4 (13)°, *viz*. it is smaller than that of 10.6 (3)° in the free ligand (Su *et al.*, 2007). The separation between the Hg atoms in the complex molecule is 3.7919 (9) Å.

### 2. Experimental

The title compound was synthesized by the reaction of HgCl_2_ (2 mmol) and 1,2-(PCy_2_)-1,2-C_2_B_10_H_10_ (1 mmol) in dichloromethane (10 ml) under N_2_ atmosphere. The mixture was refluxed for 4 h, then a colourless solution formed. Single crystals suitable for X-ray diffraction analysis were obtained by slow evaporation of a dichloromethane-n-hexane (1:3 *v*:*v*) solution. Yield 61.7%, m. p. 453–458 K). FTIR (KBr) *v* (cm^-l^): 2989, 2966, 2930, 2872 (C—H); 2614, 2602, 2585, 2556 (B—H); 1071 (C—P).

### 3. Refinement

All H atoms were placed geometrically and treated as riding on their parent atoms, with B—H = 1.10 Å, C—H = 0.96 Å, and with *U*_iso_(H) = 1.2*U*_eq_(C, B). The C–C interatomic distances within the cyclohexyl rings were restrained to be similar (SADI restraints with default standard deviations).

### Crystal data

**Table:**

[Hg_2_Cl_4_(C_26_H_54_B_10_P_2_)]	*F*(000) = 1036
*M**_r_* = 1079.71	*D*_x_ = 1.804 Mg m^−^^3^
Monoclinic, *P*2_1_	Mo *K*α radiation, λ = 0.71073 Å
Hall symbol: P 2yb	Cell parameters from 7522 reflections
*a* = 10.2233 (11) Å	θ = 2.3–27.2°
*b* = 16.6581 (18) Å	µ = 8.08 mm^−^^1^
*c* = 11.7146 (15) Å	*T* = 298 K
β = 95.000 (1)°	Block, yellow
*V* = 1987.4 (4) Å^3^	0.31 × 0.28 × 0.26 mm
*Z* = 2	

### Data collection

**Table:**

Bruker SMART1000 CCD diffractometer	6455 independent reflections
Radiation source: fine-focus sealed tube	5998 reflections with *I* > 2σ(*I*)
Graphite monochromator	*R*_int_ = 0.047
phi and ω scans	θ_max_ = 25.0°, θ_min_ = 2.0°
Absorption correction: multi-scan (*SADABS*; Sheldrick, 1996)	*h* = −12→7
*T*_min_ = 0.188, *T*_max_ = 0.228	*k* = −19→18
10374 measured reflections	*l* = −11→13

### Refinement

**Table:**

Refinement on *F*^2^	Secondary atom site location: difference Fourier map
Least-squares matrix: full	Hydrogen site location: inferred from neighbouring sites
*R*[*F*^2^ > 2σ(*F*^2^)] = 0.049	H-atom parameters constrained
*wR*(*F*^2^) = 0.148	*w* = 1/[σ^2^(*F*_o_^2^) + (0.0892*P*)^2^ + 12.4468*P*] where *P* = (*F*_o_^2^ + 2*F*_c_^2^)/3
*S* = 1.08	(Δ/σ)_max_ < 0.001
6455 reflections	Δρ_max_ = 1.33 e Å^−^^3^
397 parameters	Δρ_min_ = −1.45 e Å^−^^3^
61 restraints	Absolute structure: Flack (1983), 2819 Friedel pairs
Primary atom site location: structure-invariant direct methods	Absolute structure parameter: −0.010 (11)

### Fractional atomic coordinates and isotropic or equivalent isotropic displacement parameters (Å^2^)

**Table:**

	*x*	*y*	*z*	*U*_iso_*/*U*_eq_	
Hg1	0.21194 (5)	0.33141 (3)	0.42706 (4)	0.03789 (15)	
Hg2	0.19581 (7)	0.18076 (4)	0.66809 (6)	0.0600 (2)	
P1	0.3218 (3)	0.4632 (2)	0.4754 (3)	0.0306 (7)	
P2	0.1709 (3)	0.36694 (19)	0.2179 (3)	0.0280 (6)	
Cl1	0.3605 (4)	0.2141 (2)	0.4836 (4)	0.0531 (10)	
Cl2	0.0243 (3)	0.2714 (2)	0.5197 (3)	0.0506 (9)	
Cl3	0.1506 (5)	0.0496 (3)	0.6128 (6)	0.0807 (16)	
Cl4	0.2425 (7)	0.2817 (4)	0.8022 (5)	0.0906 (17)	
B1	0.1821 (15)	0.5456 (9)	0.2675 (14)	0.037 (3)	
H1	0.0894	0.5385	0.3075	0.044*	
B2	0.3086 (16)	0.6146 (10)	0.3213 (16)	0.045 (4)	
H2	0.2953	0.6549	0.3938	0.054*	
B3	0.4616 (15)	0.5675 (10)	0.3004 (15)	0.041 (4)	
H3	0.5495	0.5773	0.3597	0.049*	
B4	0.4220 (13)	0.4721 (10)	0.2368 (14)	0.038 (3)	
H4	0.4811	0.4183	0.2589	0.046*	
B5	0.3457 (15)	0.4900 (11)	0.0961 (14)	0.041 (4)	
H5	0.3594	0.4491	0.0244	0.050*	
B6	0.1937 (16)	0.5384 (10)	0.1145 (12)	0.036 (3)	
H6	0.1061	0.5304	0.0542	0.044*	
B7	0.2274 (19)	0.6321 (11)	0.1834 (17)	0.051 (4)	
H7	0.1615	0.6839	0.1685	0.062*	
B8	0.4004 (19)	0.6428 (12)	0.2007 (17)	0.054 (5)	
H8	0.4480	0.7018	0.1955	0.064*	
B9	0.4747 (17)	0.5544 (12)	0.1493 (16)	0.051 (4)	
H9	0.5699	0.5548	0.1121	0.061*	
B10	0.3297 (19)	0.5969 (12)	0.0774 (15)	0.050 (4)	
H10	0.3308	0.6261	−0.0068	0.060*	
C1	0.3301 (12)	0.5145 (8)	0.3336 (12)	0.036 (3)	
C2	0.2549 (12)	0.4663 (8)	0.2035 (12)	0.035 (3)	
C3	−0.0013 (10)	0.3873 (8)	0.1601 (9)	0.031 (3)	
H3A	−0.0159	0.4446	0.1727	0.037*	
C4	−0.0280 (12)	0.3739 (10)	0.0312 (10)	0.048 (4)	
H4A	−0.0184	0.3174	0.0138	0.057*	
H4B	0.0353	0.4038	−0.0090	0.057*	
C5	−0.1670 (12)	0.4016 (13)	−0.0093 (15)	0.069 (5)	
H5A	−0.1744	0.4590	0.0032	0.083*	
H5B	−0.1838	0.3916	−0.0908	0.083*	
C6	−0.2687 (15)	0.3577 (13)	0.0549 (12)	0.070 (6)	
H6A	−0.3555	0.3784	0.0311	0.084*	
H6B	−0.2675	0.3010	0.0361	0.084*	
C7	−0.2404 (12)	0.3684 (10)	0.1837 (12)	0.049 (4)	
H7A	−0.3029	0.3368	0.2224	0.059*	
H7B	−0.2530	0.4243	0.2029	0.059*	
C8	−0.1014 (10)	0.3431 (9)	0.2273 (11)	0.038 (3)	
H8A	−0.0916	0.2856	0.2181	0.046*	
H8B	−0.0857	0.3556	0.3082	0.046*	
C9	0.2420 (10)	0.2883 (8)	0.1285 (10)	0.033 (3)	
H9A	0.2272	0.3029	0.0473	0.040*	
C10	0.1644 (16)	0.2125 (8)	0.1513 (16)	0.055 (4)	
H10A	0.0726	0.2212	0.1259	0.066*	
H10B	0.1704	0.2025	0.2331	0.066*	
C11	0.2136 (16)	0.1385 (10)	0.0905 (18)	0.068 (5)	
H11A	0.1642	0.0916	0.1103	0.081*	
H11B	0.2007	0.1459	0.0081	0.081*	
C12	0.3596 (16)	0.1260 (10)	0.1268 (19)	0.074 (6)	
H12A	0.3719	0.1158	0.2086	0.089*	
H12B	0.3918	0.0799	0.0873	0.089*	
C13	0.4365 (15)	0.2009 (8)	0.0977 (16)	0.057 (4)	
H13A	0.5292	0.1925	0.1195	0.069*	
H13B	0.4255	0.2101	0.0157	0.069*	
C14	0.3894 (12)	0.2744 (9)	0.1599 (13)	0.047 (3)	
H14A	0.4384	0.3212	0.1391	0.056*	
H14B	0.4054	0.2666	0.2420	0.056*	
C15	0.2226 (12)	0.5304 (8)	0.5569 (9)	0.038 (3)	
H15	0.1670	0.5620	0.5013	0.045*	
C16	0.3044 (15)	0.5906 (9)	0.6347 (13)	0.051 (4)	
H16A	0.3656	0.5612	0.6871	0.061*	
H16B	0.3548	0.6247	0.5877	0.061*	
C17	0.2156 (17)	0.6432 (11)	0.7037 (14)	0.068 (5)	
H17A	0.2694	0.6789	0.7535	0.081*	
H17B	0.1589	0.6757	0.6517	0.081*	
C18	0.1312 (17)	0.5903 (11)	0.7764 (15)	0.073 (6)	
H18A	0.1877	0.5587	0.8298	0.087*	
H18B	0.0765	0.6240	0.8201	0.087*	
C19	0.0442 (16)	0.5343 (12)	0.6979 (15)	0.065 (5)	
H19A	−0.0083	0.5007	0.7439	0.078*	
H19B	−0.0148	0.5659	0.6465	0.078*	
C20	0.1303 (16)	0.4813 (10)	0.6281 (14)	0.053 (4)	
H20A	0.0742	0.4479	0.5768	0.064*	
H20B	0.1827	0.4462	0.6798	0.064*	
C21	0.4896 (11)	0.4527 (8)	0.5507 (9)	0.036 (3)	
H21	0.5241	0.5062	0.5708	0.043*	
C22	0.4702 (13)	0.4067 (9)	0.6606 (10)	0.043 (3)	
H22A	0.4319	0.3547	0.6411	0.052*	
H22B	0.4093	0.4359	0.7042	0.052*	
C23	0.5996 (14)	0.3947 (12)	0.7349 (13)	0.060 (5)	
H23A	0.6348	0.4463	0.7606	0.071*	
H23B	0.5843	0.3630	0.8019	0.071*	
C24	0.6978 (15)	0.3517 (11)	0.6648 (13)	0.063 (5)	
H24A	0.7812	0.3463	0.7104	0.075*	
H24B	0.6656	0.2983	0.6450	0.075*	
C25	0.7176 (14)	0.3982 (12)	0.5562 (14)	0.063 (5)	
H25A	0.7798	0.3698	0.5129	0.075*	
H25B	0.7541	0.4505	0.5763	0.075*	
C26	0.5882 (11)	0.4086 (10)	0.4818 (13)	0.046 (3)	
H26A	0.5534	0.3565	0.4580	0.055*	
H26B	0.6030	0.4392	0.4137	0.055*	

### Atomic displacement parameters (Å^2^)

**Table:**

	*U*^11^	*U*^22^	*U*^33^	*U*^12^	*U*^13^	*U*^23^
Hg1	0.0419 (3)	0.0364 (3)	0.0351 (3)	−0.0001 (2)	0.00177 (18)	0.0050 (2)
Hg2	0.0627 (4)	0.0638 (4)	0.0535 (4)	0.0074 (3)	0.0045 (3)	0.0143 (3)
P1	0.0287 (16)	0.0342 (18)	0.0284 (16)	0.0006 (13)	−0.0008 (13)	−0.0008 (12)
P2	0.0224 (13)	0.0296 (15)	0.0315 (17)	−0.0018 (11)	0.0001 (12)	0.0029 (12)
Cl1	0.049 (2)	0.047 (2)	0.064 (3)	0.0168 (17)	0.0111 (18)	0.0181 (18)
Cl2	0.0351 (16)	0.061 (2)	0.056 (2)	0.0028 (15)	0.0052 (15)	0.0203 (17)
Cl3	0.052 (2)	0.055 (3)	0.137 (5)	0.001 (2)	0.024 (3)	0.014 (3)
Cl4	0.125 (5)	0.095 (4)	0.052 (3)	0.002 (4)	0.007 (3)	0.002 (3)
B1	0.035 (7)	0.031 (8)	0.044 (9)	0.002 (6)	−0.002 (6)	−0.003 (6)
B2	0.041 (8)	0.034 (8)	0.058 (11)	−0.003 (6)	−0.012 (8)	0.000 (7)
B3	0.035 (7)	0.043 (9)	0.044 (9)	−0.011 (6)	−0.001 (6)	−0.011 (7)
B4	0.024 (6)	0.042 (8)	0.047 (9)	−0.005 (6)	−0.003 (6)	−0.004 (7)
B5	0.037 (8)	0.052 (10)	0.035 (8)	−0.015 (7)	0.002 (6)	0.006 (7)
B6	0.046 (8)	0.046 (9)	0.016 (6)	−0.002 (7)	−0.006 (6)	0.009 (6)
B7	0.053 (9)	0.039 (9)	0.059 (11)	−0.002 (8)	−0.015 (8)	0.007 (8)
B8	0.053 (10)	0.052 (11)	0.055 (11)	−0.019 (8)	0.003 (8)	0.019 (8)
B9	0.041 (8)	0.064 (12)	0.048 (10)	−0.022 (8)	0.006 (7)	−0.002 (8)
B10	0.054 (10)	0.056 (11)	0.039 (10)	−0.019 (8)	−0.001 (8)	0.018 (8)
C1	0.028 (6)	0.033 (7)	0.046 (8)	−0.001 (5)	0.004 (5)	0.004 (5)
C2	0.029 (6)	0.033 (7)	0.042 (8)	−0.004 (5)	0.005 (5)	0.002 (5)
C3	0.025 (5)	0.044 (7)	0.021 (6)	−0.005 (5)	−0.011 (5)	0.002 (5)
C4	0.038 (7)	0.070 (10)	0.034 (8)	−0.008 (7)	−0.002 (6)	−0.001 (7)
C5	0.050 (9)	0.098 (15)	0.053 (11)	−0.005 (9)	−0.027 (8)	0.017 (10)
C6	0.036 (8)	0.099 (16)	0.072 (13)	−0.006 (8)	−0.013 (8)	−0.002 (10)
C7	0.027 (6)	0.058 (9)	0.063 (10)	−0.006 (6)	0.002 (6)	0.007 (7)
C8	0.029 (5)	0.048 (9)	0.038 (7)	−0.002 (6)	0.000 (5)	0.010 (6)
C9	0.038 (6)	0.051 (8)	0.011 (5)	−0.001 (6)	0.009 (5)	−0.007 (5)
C10	0.053 (9)	0.046 (9)	0.069 (11)	−0.014 (7)	0.021 (8)	−0.028 (8)
C11	0.084 (13)	0.036 (9)	0.087 (14)	−0.002 (9)	0.025 (11)	−0.023 (9)
C12	0.091 (14)	0.047 (11)	0.088 (15)	0.027 (10)	0.030 (12)	−0.011 (9)
C13	0.049 (8)	0.055 (10)	0.070 (12)	0.017 (7)	0.016 (8)	−0.020 (8)
C14	0.038 (7)	0.057 (10)	0.045 (8)	0.012 (6)	0.004 (6)	−0.010 (7)
C15	0.050 (7)	0.052 (8)	0.011 (5)	0.010 (6)	0.001 (5)	−0.010 (5)
C16	0.058 (9)	0.049 (9)	0.043 (9)	0.008 (7)	−0.005 (7)	−0.015 (7)
C17	0.077 (12)	0.065 (11)	0.057 (11)	0.028 (9)	−0.019 (9)	−0.028 (9)
C18	0.078 (12)	0.091 (15)	0.048 (10)	0.034 (11)	−0.002 (9)	−0.025 (10)
C19	0.058 (10)	0.089 (14)	0.050 (10)	0.011 (9)	0.017 (8)	−0.017 (9)
C20	0.062 (9)	0.054 (9)	0.047 (9)	0.012 (8)	0.030 (8)	−0.004 (7)
C21	0.030 (6)	0.048 (8)	0.028 (7)	0.003 (5)	−0.006 (5)	0.004 (5)
C22	0.049 (8)	0.051 (9)	0.027 (7)	0.005 (6)	−0.009 (6)	−0.005 (6)
C23	0.067 (10)	0.070 (11)	0.037 (9)	0.011 (9)	−0.023 (8)	−0.004 (7)
C24	0.050 (9)	0.064 (12)	0.072 (12)	0.021 (7)	−0.013 (8)	0.009 (8)
C25	0.036 (8)	0.068 (12)	0.082 (13)	0.014 (8)	−0.006 (8)	0.011 (10)
C26	0.037 (7)	0.055 (9)	0.046 (9)	0.003 (6)	0.000 (6)	−0.013 (7)

### Geometric parameters (Å, º)

**Table:**

Hg1—Cl2	2.494 (3)	C5—H5A	0.9700
Hg1—P1	2.508 (3)	C5—H5B	0.9700
Hg1—P2	2.520 (3)	C6—C7	1.521 (14)
Hg1—Cl1	2.528 (4)	C6—H6A	0.9700
Hg2—Cl3	2.313 (6)	C6—H6B	0.9700
Hg2—Cl4	2.322 (6)	C7—C8	1.528 (12)
Hg2—Cl2	2.802 (4)	C7—H7A	0.9700
Hg2—Cl1	2.906 (4)	C7—H7B	0.9700
P1—C15	1.834 (13)	C8—H8A	0.9700
P1—C21	1.867 (12)	C8—H8B	0.9700
P1—C1	1.876 (14)	C9—C10	1.528 (13)
P2—C3	1.862 (11)	C9—C14	1.537 (13)
P2—C9	1.864 (13)	C9—H9A	0.9800
P2—C2	1.878 (13)	C10—C11	1.531 (13)
B1—C1	1.718 (19)	C10—H10A	0.9700
B1—C2	1.72 (2)	C10—H10B	0.9700
B1—B2	1.80 (2)	C11—C12	1.530 (15)
B1—B6	1.81 (2)	C11—H11A	0.9700
B1—B7	1.83 (2)	C11—H11B	0.9700
B1—H1	1.1000	C12—C13	1.528 (14)
B2—C1	1.69 (2)	C12—H12A	0.9700
B2—B7	1.77 (2)	C12—H12B	0.9700
B2—B3	1.79 (2)	C13—C14	1.523 (13)
B2—B8	1.82 (3)	C13—H13A	0.9700
B2—H2	1.1000	C13—H13B	0.9700
B3—C1	1.682 (19)	C14—H14A	0.9700
B3—B4	1.79 (2)	C14—H14B	0.9700
B3—B8	1.79 (3)	C15—C20	1.547 (13)
B3—B9	1.80 (2)	C15—C16	1.549 (13)
B3—H3	1.1000	C15—H15	0.9800
B4—C1	1.69 (2)	C16—C17	1.541 (14)
B4—C2	1.722 (18)	C16—H16A	0.9700
B4—B5	1.79 (2)	C16—H16B	0.9700
B4—B9	1.82 (2)	C17—C18	1.540 (15)
B4—H4	1.1000	C17—H17A	0.9700
B5—C2	1.68 (2)	C17—H17B	0.9700
B5—B9	1.77 (2)	C18—C19	1.538 (14)
B5—B6	1.78 (2)	C18—H18A	0.9700
B5—B10	1.80 (3)	C18—H18B	0.9700
B5—H5	1.1000	C19—C20	1.531 (13)
B6—C2	1.676 (19)	C19—H19A	0.9700
B6—B7	1.78 (2)	C19—H19B	0.9700
B6—B10	1.78 (2)	C20—H20A	0.9700
B6—H6	1.1000	C20—H20B	0.9700
B7—B8	1.77 (3)	C21—C22	1.526 (13)
B7—B10	1.79 (3)	C21—C26	1.532 (13)
B7—H7	1.1000	C21—H21	0.9800
B8—B10	1.73 (3)	C22—C23	1.532 (13)
B8—B9	1.79 (3)	C22—H22A	0.9700
B8—H8	1.1000	C22—H22B	0.9700
B9—B10	1.79 (3)	C23—C24	1.529 (14)
B9—H9	1.1000	C23—H23A	0.9700
B10—H10	1.1000	C23—H23B	0.9700
C1—C2	1.832 (19)	C24—C25	1.518 (14)
C3—C4	1.527 (12)	C24—H24A	0.9700
C3—C8	1.533 (12)	C24—H24B	0.9700
C3—H3A	0.9800	C25—C26	1.529 (13)
C4—C5	1.530 (13)	C25—H25A	0.9700
C4—H4A	0.9700	C25—H25B	0.9700
C4—H4B	0.9700	C26—H26A	0.9700
C5—C6	1.523 (14)	C26—H26B	0.9700
			
Cl2—Hg1—P1	126.79 (13)	B5—B10—H10	122.6
Cl2—Hg1—P2	116.62 (11)	B3—C1—B2	64.0 (9)
P1—Hg1—P2	92.71 (11)	B3—C1—B4	64.0 (9)
Cl2—Hg1—Cl1	92.50 (12)	B2—C1—B4	115.6 (12)
P1—Hg1—Cl1	111.75 (14)	B3—C1—B1	115.3 (11)
P2—Hg1—Cl1	118.78 (13)	B2—C1—B1	63.9 (9)
Cl3—Hg2—Cl4	153.8 (2)	B4—C1—B1	110.0 (11)
Cl3—Hg2—Cl2	103.68 (18)	B3—C1—C2	108.9 (10)
Cl4—Hg2—Cl2	96.33 (19)	B2—C1—C2	108.7 (10)
Cl3—Hg2—Cl1	94.97 (17)	B4—C1—C2	58.4 (7)
Cl4—Hg2—Cl1	105.48 (19)	B1—C1—C2	57.8 (8)
Cl2—Hg2—Cl1	78.90 (11)	B3—C1—P1	122.9 (10)
C15—P1—C21	109.8 (5)	B2—C1—P1	120.7 (10)
C15—P1—C1	104.6 (6)	B4—C1—P1	118.4 (9)
C21—P1—C1	110.5 (5)	B1—C1—P1	115.5 (9)
C15—P1—Hg1	113.3 (4)	C2—C1—P1	119.3 (8)
C21—P1—Hg1	113.6 (4)	B6—C2—B5	64.2 (9)
C1—P1—Hg1	104.6 (4)	B6—C2—B1	64.4 (9)
C3—P2—C9	109.0 (5)	B5—C2—B1	115.8 (11)
C3—P2—C2	103.4 (6)	B6—C2—B4	114.1 (11)
C9—P2—C2	111.4 (6)	B5—C2—B4	63.4 (9)
C3—P2—Hg1	117.9 (3)	B1—C2—B4	108.4 (10)
C9—P2—Hg1	110.0 (4)	B6—C2—C1	108.1 (10)
C2—P2—Hg1	104.8 (5)	B5—C2—C1	107.5 (10)
Hg1—Cl1—Hg2	88.21 (12)	B1—C2—C1	57.8 (8)
Hg1—Cl2—Hg2	91.27 (10)	B4—C2—C1	56.6 (8)
C1—B1—C2	64.4 (8)	B6—C2—P2	122.6 (9)
C1—B1—B2	57.2 (8)	B5—C2—P2	124.3 (10)
C2—B1—B2	108.6 (11)	B1—C2—P2	114.8 (9)
C1—B1—B6	107.2 (11)	B4—C2—P2	118.8 (9)
C2—B1—B6	56.6 (8)	C1—C2—P2	118.3 (8)
B2—B1—B6	106.3 (12)	C4—C3—C8	111.6 (10)
C1—B1—B7	103.1 (11)	C4—C3—P2	114.5 (8)
C2—B1—B7	103.4 (11)	C8—C3—P2	112.2 (7)
B2—B1—B7	58.5 (9)	C4—C3—H3A	105.9
B6—B1—B7	58.5 (9)	C8—C3—H3A	105.9
C1—B1—H1	122.2	P2—C3—H3A	105.9
C2—B1—H1	121.7	C3—C4—C5	110.1 (12)
B2—B1—H1	122.5	C3—C4—H4A	109.6
B6—B1—H1	123.3	C5—C4—H4A	109.6
B7—B1—H1	125.6	C3—C4—H4B	109.6
C1—B2—B7	106.6 (12)	C5—C4—H4B	109.6
C1—B2—B3	57.9 (9)	H4A—C4—H4B	108.1
B7—B2—B3	107.0 (13)	C6—C5—C4	111.0 (13)
C1—B2—B1	58.9 (8)	C6—C5—H5A	109.4
B7—B2—B1	61.4 (9)	C4—C5—H5A	109.4
B3—B2—B1	106.3 (11)	C6—C5—H5B	109.4
C1—B2—B8	104.4 (13)	C4—C5—H5B	109.4
B7—B2—B8	59.0 (11)	H5A—C5—H5B	108.0
B3—B2—B8	59.4 (10)	C7—C6—C5	110.8 (13)
B1—B2—B8	107.1 (13)	C7—C6—H6A	109.5
C1—B2—H2	124.1	C5—C6—H6A	109.5
B7—B2—H2	121.6	C7—C6—H6B	109.5
B3—B2—H2	122.9	C5—C6—H6B	109.5
B1—B2—H2	121.7	H6A—C6—H6B	108.1
B8—B2—H2	123.3	C6—C7—C8	113.0 (12)
C1—B3—B2	58.1 (9)	C6—C7—H7A	109.0
C1—B3—B4	58.2 (8)	C8—C7—H7A	109.0
B2—B3—B4	106.2 (10)	C6—C7—H7B	109.0
C1—B3—B8	106.2 (11)	C8—C7—H7B	109.0
B2—B3—B8	61.4 (10)	H7A—C7—H7B	107.8
B4—B3—B8	107.3 (12)	C7—C8—C3	109.8 (10)
C1—B3—B9	107.1 (10)	C7—C8—H8A	109.7
B2—B3—B9	109.3 (12)	C3—C8—H8A	109.7
B4—B3—B9	61.1 (9)	C7—C8—H8B	109.7
B8—B3—B9	59.7 (11)	C3—C8—H8B	109.7
C1—B3—H3	123.8	H8A—C8—H8B	108.2
B2—B3—H3	121.7	C10—C9—C14	110.3 (12)
B4—B3—H3	122.6	C10—C9—P2	104.4 (8)
B8—B3—H3	121.9	C14—C9—P2	113.2 (8)
B9—B3—H3	120.9	C10—C9—H9A	109.6
C1—B4—C2	65.0 (8)	C14—C9—H9A	109.6
C1—B4—B3	57.8 (8)	P2—C9—H9A	109.6
C2—B4—B3	109.2 (11)	C9—C10—C11	112.8 (12)
C1—B4—B5	109.1 (11)	C9—C10—H10A	109.0
C2—B4—B5	57.0 (8)	C11—C10—H10A	109.0
B3—B4—B5	107.5 (12)	C9—C10—H10B	109.0
C1—B4—B9	105.9 (12)	C11—C10—H10B	109.0
C2—B4—B9	104.6 (11)	H10A—C10—H10B	107.8
B3—B4—B9	59.9 (10)	C12—C11—C10	109.4 (14)
B5—B4—B9	58.8 (9)	C12—C11—H11A	109.8
C1—B4—H4	120.6	C10—C11—H11A	109.8
C2—B4—H4	121.4	C12—C11—H11B	109.8
B3—B4—H4	121.9	C10—C11—H11B	109.8
B5—B4—H4	122.7	H11A—C11—H11B	108.2
B9—B4—H4	124.2	C13—C12—C11	109.6 (14)
C2—B5—B9	108.9 (12)	C13—C12—H12A	109.7
C2—B5—B6	57.9 (8)	C11—C12—H12A	109.7
B9—B5—B6	108.3 (13)	C13—C12—H12B	109.7
C2—B5—B4	59.6 (8)	C11—C12—H12B	109.7
B9—B5—B4	61.6 (9)	H12A—C12—H12B	108.2
B6—B5—B4	106.2 (11)	C14—C13—C12	111.0 (13)
C2—B5—B10	106.0 (12)	C14—C13—H13A	109.4
B9—B5—B10	60.1 (10)	C12—C13—H13A	109.4
B6—B5—B10	59.7 (10)	C14—C13—H13B	109.4
B4—B5—B10	107.8 (12)	C12—C13—H13B	109.4
C2—B5—H5	122.9	H13A—C13—H13B	108.0
B9—B5—H5	120.2	C13—C14—C9	110.5 (11)
B6—B5—H5	122.9	C13—C14—H14A	109.5
B4—B5—H5	121.8	C9—C14—H14A	109.5
B10—B5—H5	122.4	C13—C14—H14B	109.5
C2—B6—B7	107.5 (11)	C9—C14—H14B	109.5
C2—B6—B5	57.9 (8)	H14A—C14—H14B	108.1
B7—B6—B5	108.4 (12)	C20—C15—C16	110.4 (11)
C2—B6—B10	106.7 (11)	C20—C15—P1	110.4 (9)
B7—B6—B10	60.4 (11)	C16—C15—P1	114.0 (9)
B5—B6—B10	60.7 (10)	C20—C15—H15	107.2
C2—B6—B1	59.0 (8)	C16—C15—H15	107.2
B7—B6—B1	61.3 (9)	P1—C15—H15	107.2
B5—B6—B1	106.5 (10)	C17—C16—C15	111.4 (12)
B10—B6—B1	109.0 (11)	C17—C16—H16A	109.4
C2—B6—H6	123.5	C15—C16—H16A	109.4
B7—B6—H6	120.9	C17—C16—H16B	109.4
B5—B6—H6	122.5	C15—C16—H16B	109.4
B10—B6—H6	121.2	H16A—C16—H16B	108.0
B1—B6—H6	121.6	C18—C17—C16	110.4 (14)
B8—B7—B2	61.9 (10)	C18—C17—H17A	109.6
B8—B7—B6	107.0 (14)	C16—C17—H17A	109.6
B2—B7—B6	109.1 (12)	C18—C17—H17B	109.6
B8—B7—B10	58.3 (11)	C16—C17—H17B	109.6
B2—B7—B10	108.8 (13)	H17A—C17—H17B	108.1
B6—B7—B10	60.0 (10)	C19—C18—C17	109.8 (15)
B8—B7—B1	108.4 (12)	C19—C18—H18A	109.7
B2—B7—B1	60.1 (9)	C17—C18—H18A	109.7
B6—B7—B1	60.3 (9)	C19—C18—H18B	109.7
B10—B7—B1	107.9 (12)	C17—C18—H18B	109.7
B8—B7—H7	122.0	H18A—C18—H18B	108.2
B2—B7—H7	120.3	C20—C19—C18	109.9 (14)
B6—B7—H7	121.7	C20—C19—H19A	109.7
B10—B7—H7	122.2	C18—C19—H19A	109.7
B1—B7—H7	121.6	C20—C19—H19B	109.7
B10—B8—B7	61.4 (11)	C18—C19—H19B	109.7
B10—B8—B9	61.0 (12)	H19A—C19—H19B	108.2
B7—B8—B9	109.2 (13)	C19—C20—C15	112.9 (13)
B10—B8—B3	109.3 (13)	C19—C20—H20A	109.0
B7—B8—B3	107.0 (12)	C15—C20—H20A	109.0
B9—B8—B3	60.5 (11)	C19—C20—H20B	109.0
B10—B8—B2	109.1 (12)	C15—C20—H20B	109.0
B7—B8—B2	59.1 (10)	H20A—C20—H20B	107.8
B9—B8—B2	108.2 (12)	C22—C21—C26	110.1 (11)
B3—B8—B2	59.2 (9)	C22—C21—P1	105.3 (8)
B10—B8—H8	120.1	C26—C21—P1	114.5 (8)
B7—B8—H8	121.8	C22—C21—H21	108.9
B9—B8—H8	120.9	C26—C21—H21	108.9
B3—B8—H8	122.0	P1—C21—H21	108.9
B2—B8—H8	122.2	C21—C22—C23	112.1 (12)
B5—B9—B8	107.0 (13)	C21—C22—H22A	109.2
B5—B9—B10	60.8 (10)	C23—C22—H22A	109.2
B8—B9—B10	58.1 (11)	C21—C22—H22B	109.2
B5—B9—B3	107.6 (11)	C23—C22—H22B	109.2
B8—B9—B3	59.9 (10)	H22A—C22—H22B	107.9
B10—B9—B3	106.6 (13)	C24—C23—C22	109.3 (12)
B5—B9—B4	59.6 (9)	C24—C23—H23A	109.8
B8—B9—B4	106.0 (12)	C22—C23—H23A	109.8
B10—B9—B4	106.8 (11)	C24—C23—H23B	109.8
B3—B9—B4	59.1 (9)	C22—C23—H23B	109.8
B5—B9—H9	121.5	H23A—C23—H23B	108.3
B8—B9—H9	123.1	C25—C24—C23	110.7 (14)
B10—B9—H9	122.5	C25—C24—H24A	109.5
B3—B9—H9	122.2	C23—C24—H24A	109.5
B4—B9—H9	122.9	C25—C24—H24B	109.5
B8—B10—B6	108.3 (12)	C23—C24—H24B	109.5
B8—B10—B9	60.9 (11)	H24A—C24—H24B	108.1
B6—B10—B9	107.5 (11)	C24—C25—C26	111.3 (13)
B8—B10—B7	60.3 (11)	C24—C25—H25A	109.4
B6—B10—B7	59.6 (10)	C26—C25—H25A	109.4
B9—B10—B7	108.3 (12)	C24—C25—H25B	109.4
B8—B10—B5	107.9 (12)	C26—C25—H25B	109.4
B6—B10—B5	59.6 (9)	H25A—C25—H25B	108.0
B9—B10—B5	59.2 (10)	C25—C26—C21	109.3 (11)
B7—B10—B5	106.9 (11)	C25—C26—H26A	109.8
B8—B10—H10	121.0	C21—C26—H26A	109.8
B6—B10—H10	122.0	C25—C26—H26B	109.8
B9—B10—H10	121.7	C21—C26—H26B	109.8
B7—B10—H10	122.0	H26A—C26—H26B	108.3
			
Cl2—Hg1—P1—C15	−18.0 (5)	B5—B9—B10—B7	99.2 (12)
P2—Hg1—P1—C15	108.5 (5)	B8—B9—B10—B7	−38.7 (12)
Cl1—Hg1—P1—C15	−128.9 (5)	B3—B9—B10—B7	−2.0 (16)
Cl2—Hg1—P1—C21	108.1 (4)	B4—B9—B10—B7	59.9 (15)
P2—Hg1—P1—C21	−125.4 (4)	B8—B9—B10—B5	−137.9 (13)
Cl1—Hg1—P1—C21	−2.9 (4)	B3—B9—B10—B5	−101.2 (13)
Cl2—Hg1—P1—C1	−131.4 (4)	B4—B9—B10—B5	−39.3 (11)
P2—Hg1—P1—C1	−4.8 (4)	B2—B7—B10—B8	−37.3 (12)
Cl1—Hg1—P1—C1	117.7 (4)	B6—B7—B10—B8	−139.0 (13)
Cl2—Hg1—P2—C3	24.7 (5)	B1—B7—B10—B8	−101.0 (13)
P1—Hg1—P2—C3	−109.3 (5)	B8—B7—B10—B6	139.0 (13)
Cl1—Hg1—P2—C3	134.0 (5)	B2—B7—B10—B6	101.6 (13)
Cl2—Hg1—P2—C9	−101.2 (4)	B1—B7—B10—B6	38.0 (10)
P1—Hg1—P2—C9	124.9 (4)	B8—B7—B10—B9	39.0 (12)
Cl1—Hg1—P2—C9	8.2 (4)	B2—B7—B10—B9	1.6 (16)
Cl2—Hg1—P2—C2	139.0 (4)	B6—B7—B10—B9	−100.0 (12)
P1—Hg1—P2—C2	5.0 (4)	B1—B7—B10—B9	−62.0 (15)
Cl1—Hg1—P2—C2	−111.7 (4)	B8—B7—B10—B5	101.3 (13)
Cl2—Hg1—Cl1—Hg2	−23.46 (14)	B2—B7—B10—B5	64.0 (15)
P1—Hg1—Cl1—Hg2	108.06 (13)	B6—B7—B10—B5	−37.6 (10)
P2—Hg1—Cl1—Hg2	−145.84 (10)	B1—B7—B10—B5	0.4 (15)
Cl3—Hg2—Cl1—Hg1	124.14 (18)	C2—B5—B10—B8	64.9 (15)
Cl4—Hg2—Cl1—Hg1	−72.4 (2)	B9—B5—B10—B8	−38.0 (12)
Cl2—Hg2—Cl1—Hg1	21.15 (13)	B6—B5—B10—B8	101.2 (14)
P1—Hg1—Cl2—Hg2	−95.34 (14)	B4—B5—B10—B8	2.4 (16)
P2—Hg1—Cl2—Hg2	148.51 (10)	C2—B5—B10—B6	−36.2 (10)
Cl1—Hg1—Cl2—Hg2	24.38 (15)	B9—B5—B10—B6	−139.2 (12)
Cl3—Hg2—Cl2—Hg1	−113.91 (17)	B4—B5—B10—B6	−98.7 (11)
Cl4—Hg2—Cl2—Hg1	83.1 (2)	C2—B5—B10—B9	102.9 (12)
Cl1—Hg2—Cl2—Hg1	−21.44 (13)	B6—B5—B10—B9	139.2 (12)
C2—B1—B2—C1	−40.6 (10)	B4—B5—B10—B9	40.4 (11)
B6—B1—B2—C1	−100.2 (11)	C2—B5—B10—B7	1.4 (15)
B7—B1—B2—C1	−135.3 (13)	B9—B5—B10—B7	−101.5 (13)
C1—B1—B2—B7	135.3 (13)	B6—B5—B10—B7	37.6 (11)
C2—B1—B2—B7	94.6 (13)	B4—B5—B10—B7	−61.1 (14)
B6—B1—B2—B7	35.0 (11)	B4—B3—C1—B2	−140.6 (12)
C1—B1—B2—B3	34.4 (11)	B8—B3—C1—B2	−39.9 (12)
C2—B1—B2—B3	−6.2 (15)	B9—B3—C1—B2	−102.4 (14)
B6—B1—B2—B3	−65.8 (14)	B2—B3—C1—B4	140.6 (12)
B7—B1—B2—B3	−100.8 (14)	B8—B3—C1—B4	100.7 (13)
C1—B1—B2—B8	96.8 (13)	B9—B3—C1—B4	38.1 (12)
C2—B1—B2—B8	56.1 (14)	B2—B3—C1—B1	39.6 (12)
B6—B1—B2—B8	−3.5 (15)	B4—B3—C1—B1	−101.0 (13)
B7—B1—B2—B8	−38.5 (12)	B8—B3—C1—B1	−0.3 (16)
B7—B2—B3—C1	−99.3 (12)	B9—B3—C1—B1	−62.9 (15)
B1—B2—B3—C1	−34.9 (11)	B2—B3—C1—C2	102.1 (11)
B8—B2—B3—C1	−135.4 (12)	B4—B3—C1—C2	−38.5 (10)
C1—B2—B3—B4	34.2 (11)	B8—B3—C1—C2	62.2 (13)
B7—B2—B3—B4	−65.1 (14)	B9—B3—C1—C2	−0.3 (15)
B1—B2—B3—B4	−0.7 (16)	B2—B3—C1—P1	−111.1 (13)
B8—B2—B3—B4	−101.3 (13)	B4—B3—C1—P1	108.3 (12)
C1—B2—B3—B8	135.4 (12)	B8—B3—C1—P1	−151.0 (11)
B7—B2—B3—B8	36.1 (12)	B9—B3—C1—P1	146.4 (11)
B1—B2—B3—B8	100.6 (13)	B7—B2—C1—B3	100.0 (14)
C1—B2—B3—B9	98.6 (12)	B1—B2—C1—B3	140.1 (12)
B7—B2—B3—B9	−0.7 (15)	B8—B2—C1—B3	38.6 (11)
B1—B2—B3—B9	63.7 (15)	B7—B2—C1—B4	60.7 (15)
B8—B2—B3—B9	−36.9 (12)	B3—B2—C1—B4	−39.3 (11)
B2—B3—B4—C1	−34.2 (11)	B1—B2—C1—B4	100.9 (12)
B8—B3—B4—C1	−98.6 (12)	B8—B2—C1—B4	−0.7 (15)
B9—B3—B4—C1	−137.6 (12)	B7—B2—C1—B1	−40.2 (11)
C1—B3—B4—C2	41.5 (11)	B3—B2—C1—B1	−140.1 (12)
B2—B3—B4—C2	7.3 (16)	B8—B2—C1—B1	−101.6 (12)
B8—B3—B4—C2	−57.1 (15)	B7—B2—C1—C2	−2.5 (15)
B9—B3—B4—C2	−96.1 (12)	B3—B2—C1—C2	−102.4 (11)
C1—B3—B4—B5	101.9 (11)	B1—B2—C1—C2	37.7 (10)
B2—B3—B4—B5	67.7 (14)	B8—B2—C1—C2	−63.9 (12)
B8—B3—B4—B5	3.3 (15)	B7—B2—C1—P1	−145.7 (11)
B9—B3—B4—B5	−35.7 (11)	B3—B2—C1—P1	114.4 (11)
C1—B3—B4—B9	137.6 (12)	B1—B2—C1—P1	−105.5 (11)
B2—B3—B4—B9	103.4 (13)	B8—B2—C1—P1	153.0 (10)
B8—B3—B4—B9	39.0 (12)	C2—B4—C1—B3	−136.3 (11)
C1—B4—B5—C2	−40.7 (10)	B5—B4—C1—B3	−99.1 (13)
B3—B4—B5—C2	−102.0 (11)	B9—B4—C1—B3	−37.3 (11)
B9—B4—B5—C2	−138.1 (13)	C2—B4—C1—B2	−97.0 (12)
C1—B4—B5—B9	97.4 (13)	B3—B4—C1—B2	39.3 (11)
C2—B4—B5—B9	138.1 (13)	B5—B4—C1—B2	−59.9 (14)
B3—B4—B5—B9	36.2 (11)	B9—B4—C1—B2	1.9 (14)
C1—B4—B5—B6	−5.1 (15)	C2—B4—C1—B1	−27.2 (10)
C2—B4—B5—B6	35.7 (10)	B3—B4—C1—B1	109.1 (12)
B3—B4—B5—B6	−66.3 (13)	B5—B4—C1—B1	10.0 (14)
B9—B4—B5—B6	−102.4 (13)	B9—B4—C1—B1	71.8 (12)
C1—B4—B5—B10	57.7 (14)	B3—B4—C1—C2	136.3 (11)
C2—B4—B5—B10	98.4 (13)	B5—B4—C1—C2	37.2 (9)
B3—B4—B5—B10	−3.5 (14)	B9—B4—C1—C2	99.0 (11)
B9—B4—B5—B10	−39.7 (12)	C2—B4—C1—P1	108.7 (10)
B9—B5—B6—C2	−101.2 (12)	B3—B4—C1—P1	−115.0 (11)
B4—B5—B6—C2	−36.4 (10)	B5—B4—C1—P1	145.9 (10)
B10—B5—B6—C2	−137.9 (12)	B9—B4—C1—P1	−152.3 (9)
C2—B5—B6—B7	99.5 (12)	C2—B1—C1—B3	97.2 (12)
B9—B5—B6—B7	−1.7 (15)	B2—B1—C1—B3	−39.6 (12)
B4—B5—B6—B7	63.1 (14)	B6—B1—C1—B3	59.1 (14)
B10—B5—B6—B7	−38.4 (11)	B7—B1—C1—B3	−1.5 (15)
C2—B5—B6—B10	137.9 (12)	C2—B1—C1—B2	136.8 (11)
B9—B5—B6—B10	36.6 (11)	B6—B1—C1—B2	98.7 (12)
B4—B5—B6—B10	101.5 (12)	B7—B1—C1—B2	38.0 (11)
C2—B5—B6—B1	35.0 (10)	C2—B1—C1—B4	27.4 (10)
B9—B5—B6—B1	−66.3 (14)	B2—B1—C1—B4	−109.5 (12)
B4—B5—B6—B1	−1.4 (14)	B6—B1—C1—B4	−10.8 (14)
B10—B5—B6—B1	−102.9 (12)	B7—B1—C1—B4	−71.4 (13)
C1—B1—B6—C2	41.8 (10)	B2—B1—C1—C2	−136.8 (11)
B2—B1—B6—C2	101.8 (11)	B6—B1—C1—C2	−38.1 (9)
B7—B1—B6—C2	136.9 (12)	B7—B1—C1—C2	−98.8 (11)
C1—B1—B6—B7	−95.1 (12)	C2—B1—C1—P1	−109.9 (10)
C2—B1—B6—B7	−136.9 (12)	B2—B1—C1—P1	113.3 (12)
B2—B1—B6—B7	−35.1 (11)	B6—B1—C1—P1	−148.0 (9)
C1—B1—B6—B5	7.3 (14)	B7—B1—C1—P1	151.4 (10)
C2—B1—B6—B5	−34.5 (10)	C15—P1—C1—B3	100.8 (11)
B2—B1—B6—B5	67.3 (13)	C21—P1—C1—B3	−17.3 (13)
B7—B1—B6—B5	102.4 (12)	Hg1—P1—C1—B3	−139.9 (10)
C1—B1—B6—B10	−56.7 (14)	C15—P1—C1—B2	23.7 (11)
C2—B1—B6—B10	−98.5 (12)	C21—P1—C1—B2	−94.4 (11)
B2—B1—B6—B10	3.3 (15)	Hg1—P1—C1—B2	143.0 (9)
B7—B1—B6—B10	38.4 (12)	C15—P1—C1—B4	176.6 (9)
C1—B2—B7—B8	−97.1 (13)	C21—P1—C1—B4	58.5 (11)
B3—B2—B7—B8	−36.3 (12)	Hg1—P1—C1—B4	−64.1 (9)
B1—B2—B7—B8	−136.0 (13)	C15—P1—C1—B1	−49.9 (11)
C1—B2—B7—B6	2.5 (17)	C21—P1—C1—B1	−167.9 (9)
B3—B2—B7—B6	63.2 (15)	Hg1—P1—C1—B1	69.5 (10)
B1—B2—B7—B6	−36.4 (12)	C15—P1—C1—C2	−115.7 (9)
B8—B2—B7—B6	99.6 (15)	C21—P1—C1—C2	126.2 (9)
C1—B2—B7—B10	−61.3 (15)	Hg1—P1—C1—C2	3.6 (9)
B3—B2—B7—B10	−0.6 (15)	B7—B6—C2—B5	−101.2 (13)
B1—B2—B7—B10	−100.2 (13)	B10—B6—C2—B5	−37.6 (12)
B8—B2—B7—B10	35.8 (12)	B1—B6—C2—B5	−140.1 (11)
C1—B2—B7—B1	39.0 (11)	B7—B6—C2—B1	38.9 (11)
B3—B2—B7—B1	99.7 (12)	B5—B6—C2—B1	140.1 (11)
B8—B2—B7—B1	136.0 (13)	B10—B6—C2—B1	102.5 (12)
C2—B6—B7—B8	63.9 (15)	B7—B6—C2—B4	−60.8 (15)
B5—B6—B7—B8	2.8 (15)	B5—B6—C2—B4	40.4 (11)
B10—B6—B7—B8	−35.7 (12)	B10—B6—C2—B4	2.8 (16)
B1—B6—B7—B8	101.8 (13)	B1—B6—C2—B4	−99.7 (12)
C2—B6—B7—B2	−1.5 (17)	B7—B6—C2—C1	0.0 (14)
B5—B6—B7—B2	−62.7 (15)	B5—B6—C2—C1	101.2 (10)
B10—B6—B7—B2	−101.2 (14)	B10—B6—C2—C1	63.6 (13)
B1—B6—B7—B2	36.4 (12)	B1—B6—C2—C1	−38.9 (9)
C2—B6—B7—B10	99.7 (12)	B7—B6—C2—P2	143.2 (11)
B5—B6—B7—B10	38.5 (11)	B5—B6—C2—P2	−115.7 (12)
B1—B6—B7—B10	137.6 (12)	B10—B6—C2—P2	−153.3 (11)
C2—B6—B7—B1	−37.9 (10)	B1—B6—C2—P2	104.2 (11)
B5—B6—B7—B1	−99.1 (11)	B9—B5—C2—B6	100.2 (14)
B10—B6—B7—B1	−137.6 (12)	B4—B5—C2—B6	138.6 (12)
C1—B1—B7—B8	2.8 (16)	B10—B5—C2—B6	37.1 (11)
C2—B1—B7—B8	−63.6 (15)	B9—B5—C2—B1	60.3 (16)
B2—B1—B7—B8	40.2 (13)	B6—B5—C2—B1	−40.0 (11)
B6—B1—B7—B8	−99.5 (15)	B4—B5—C2—B1	98.7 (12)
C1—B1—B7—B2	−37.4 (11)	B10—B5—C2—B1	−2.9 (15)
C2—B1—B7—B2	−103.8 (12)	B9—B5—C2—B4	−38.4 (12)
B6—B1—B7—B2	−139.7 (13)	B6—B5—C2—B4	−138.6 (12)
C1—B1—B7—B6	102.3 (11)	B10—B5—C2—B4	−101.6 (12)
C2—B1—B7—B6	35.9 (10)	B9—B5—C2—C1	−1.8 (15)
B2—B1—B7—B6	139.7 (13)	B6—B5—C2—C1	−102.0 (11)
C1—B1—B7—B10	64.5 (14)	B4—B5—C2—C1	36.6 (10)
C2—B1—B7—B10	−1.9 (14)	B10—B5—C2—C1	−65.0 (12)
B2—B1—B7—B10	101.9 (13)	B9—B5—C2—P2	−146.5 (11)
B6—B1—B7—B10	−37.9 (11)	B6—B5—C2—P2	113.3 (12)
B2—B7—B8—B10	139.4 (13)	B4—B5—C2—P2	−108.1 (12)
B6—B7—B8—B10	36.5 (12)	B10—B5—C2—P2	150.3 (10)
B1—B7—B8—B10	100.1 (14)	C1—B1—C2—B6	−135.1 (10)
B2—B7—B8—B9	100.2 (14)	B2—B1—C2—B6	−97.7 (12)
B6—B7—B8—B9	−2.8 (17)	B7—B1—C2—B6	−36.8 (10)
B10—B7—B8—B9	−39.2 (12)	C1—B1—C2—B5	−95.2 (11)
B1—B7—B8—B9	60.8 (17)	B2—B1—C2—B5	−57.8 (15)
B2—B7—B8—B3	36.3 (12)	B6—B1—C2—B5	39.9 (11)
B6—B7—B8—B3	−66.7 (16)	B7—B1—C2—B5	3.1 (14)
B10—B7—B8—B3	−103.2 (15)	C1—B1—C2—B4	−26.5 (10)
B1—B7—B8—B3	−3.1 (18)	B2—B1—C2—B4	10.9 (14)
B6—B7—B8—B2	−102.9 (13)	B6—B1—C2—B4	108.6 (11)
B10—B7—B8—B2	−139.4 (13)	B7—B1—C2—B4	71.8 (12)
B1—B7—B8—B2	−39.4 (12)	B2—B1—C2—C1	37.4 (10)
C1—B3—B8—B10	−62.8 (16)	B6—B1—C2—C1	135.1 (10)
B2—B3—B8—B10	−101.2 (14)	B7—B1—C2—C1	98.3 (11)
B4—B3—B8—B10	−1.8 (16)	C1—B1—C2—P2	109.0 (10)
B9—B3—B8—B10	37.8 (12)	B2—B1—C2—P2	146.4 (10)
C1—B3—B8—B7	2.1 (17)	B6—B1—C2—P2	−115.9 (11)
B2—B3—B8—B7	−36.2 (12)	B7—B1—C2—P2	−152.7 (9)
B4—B3—B8—B7	63.1 (16)	C1—B4—C2—B6	96.4 (12)
B9—B3—B8—B7	102.8 (14)	B3—B4—C2—B6	58.1 (15)
C1—B3—B8—B9	−100.6 (12)	B5—B4—C2—B6	−40.7 (12)
B2—B3—B8—B9	−139.0 (12)	B9—B4—C2—B6	−4.6 (15)
B4—B3—B8—B9	−39.6 (11)	C1—B4—C2—B5	137.1 (12)
C1—B3—B8—B2	38.4 (11)	B3—B4—C2—B5	98.8 (13)
B4—B3—B8—B2	99.4 (12)	B9—B4—C2—B5	36.1 (11)
B9—B3—B8—B2	139.0 (12)	C1—B4—C2—B1	26.9 (10)
C1—B2—B8—B10	63.8 (16)	B3—B4—C2—B1	−11.4 (15)
B7—B2—B8—B10	−37.2 (13)	B5—B4—C2—B1	−110.2 (12)
B3—B2—B8—B10	101.6 (15)	B9—B4—C2—B1	−74.1 (13)
B1—B2—B8—B10	2.5 (17)	B3—B4—C2—C1	−38.3 (10)
C1—B2—B8—B7	101.0 (12)	B5—B4—C2—C1	−137.1 (12)
B3—B2—B8—B7	138.8 (13)	B9—B4—C2—C1	−101.0 (12)
B1—B2—B8—B7	39.6 (12)	C1—B4—C2—P2	−106.6 (11)
C1—B2—B8—B9	−0.9 (15)	B3—B4—C2—P2	−144.8 (10)
B7—B2—B8—B9	−101.9 (14)	B5—B4—C2—P2	116.3 (12)
B3—B2—B8—B9	36.9 (11)	B9—B4—C2—P2	152.5 (10)
B1—B2—B8—B9	−62.3 (15)	B3—C1—C2—B6	−66.5 (12)
C1—B2—B8—B3	−37.8 (10)	B2—C1—C2—B6	1.6 (13)
B7—B2—B8—B3	−138.8 (13)	B4—C1—C2—B6	−107.5 (12)
B1—B2—B8—B3	−99.2 (12)	B1—C1—C2—B6	42.1 (10)
C2—B5—B9—B8	−61.4 (16)	P1—C1—C2—B6	145.4 (9)
B6—B5—B9—B8	0.0 (16)	B3—C1—C2—B5	1.3 (14)
B4—B5—B9—B8	−98.9 (13)	B2—C1—C2—B5	69.4 (13)
B10—B5—B9—B8	36.5 (12)	B4—C1—C2—B5	−39.7 (11)
C2—B5—B9—B10	−98.0 (14)	B1—C1—C2—B5	109.9 (12)
B6—B5—B9—B10	−36.5 (11)	P1—C1—C2—B5	−146.8 (10)
B4—B5—B9—B10	−135.4 (13)	B3—C1—C2—B1	−108.5 (12)
C2—B5—B9—B3	1.6 (17)	B2—C1—C2—B1	−40.5 (11)
B6—B5—B9—B3	63.0 (15)	B4—C1—C2—B1	−149.5 (12)
B4—B5—B9—B3	−35.9 (12)	P1—C1—C2—B1	103.3 (10)
B10—B5—B9—B3	99.6 (14)	B3—C1—C2—B4	41.0 (11)
C2—B5—B9—B4	37.5 (11)	B2—C1—C2—B4	109.1 (12)
B6—B5—B9—B4	98.9 (12)	B1—C1—C2—B4	149.5 (12)
B10—B5—B9—B4	135.4 (13)	P1—C1—C2—B4	−107.1 (11)
B10—B8—B9—B5	−37.7 (11)	B3—C1—C2—P2	148.5 (9)
B7—B8—B9—B5	1.7 (17)	B2—C1—C2—P2	−143.4 (10)
B3—B8—B9—B5	100.8 (12)	B4—C1—C2—P2	107.5 (11)
B2—B8—B9—B5	64.5 (15)	B1—C1—C2—P2	−103.0 (10)
B7—B8—B9—B10	39.4 (12)	P1—C1—C2—P2	0.4 (13)
B3—B8—B9—B10	138.6 (12)	C3—P2—C2—B6	−19.7 (12)
B2—B8—B9—B10	102.2 (13)	C9—P2—C2—B6	97.3 (11)
B10—B8—B9—B3	−138.6 (12)	Hg1—P2—C2—B6	−143.8 (10)
B7—B8—B9—B3	−99.1 (13)	C3—P2—C2—B5	−98.7 (11)
B2—B8—B9—B3	−36.4 (11)	C9—P2—C2—B5	18.3 (13)
B10—B8—B9—B4	−100.2 (12)	Hg1—P2—C2—B5	137.2 (10)
B7—B8—B9—B4	−60.7 (15)	C3—P2—C2—B1	54.7 (10)
B3—B8—B9—B4	38.4 (10)	C9—P2—C2—B1	171.7 (9)
B2—B8—B9—B4	2.0 (15)	Hg1—P2—C2—B1	−69.4 (9)
C1—B3—B9—B5	−0.7 (17)	C3—P2—C2—B4	−174.7 (10)
B2—B3—B9—B5	−62.2 (16)	C9—P2—C2—B4	−57.7 (12)
B4—B3—B9—B5	36.1 (12)	Hg1—P2—C2—B4	61.2 (10)
B8—B3—B9—B5	−99.8 (14)	C3—P2—C2—C1	120.0 (8)
C1—B3—B9—B8	99.1 (12)	C9—P2—C2—C1	−123.0 (9)
B2—B3—B9—B8	37.6 (11)	Hg1—P2—C2—C1	−4.1 (9)
B4—B3—B9—B8	135.9 (11)	C9—P2—C3—C4	−29.5 (12)
C1—B3—B9—B10	63.2 (15)	C2—P2—C3—C4	89.1 (11)
B2—B3—B9—B10	1.7 (15)	Hg1—P2—C3—C4	−155.8 (9)
B4—B3—B9—B10	100.0 (12)	C9—P2—C3—C8	99.1 (10)
B8—B3—B9—B10	−35.9 (11)	C2—P2—C3—C8	−142.3 (10)
C1—B3—B9—B4	−36.8 (10)	Hg1—P2—C3—C8	−27.2 (11)
B2—B3—B9—B4	−98.3 (12)	C8—C3—C4—C5	57.7 (17)
B8—B3—B9—B4	−135.9 (11)	P2—C3—C4—C5	−173.4 (11)
C1—B4—B9—B5	−103.0 (12)	C3—C4—C5—C6	−58 (2)
C2—B4—B9—B5	−35.4 (11)	C4—C5—C6—C7	56 (2)
B3—B4—B9—B5	−139.4 (13)	C5—C6—C7—C8	−55 (2)
C1—B4—B9—B8	−2.3 (14)	C6—C7—C8—C3	54.1 (18)
C2—B4—B9—B8	65.3 (14)	C4—C3—C8—C7	−55.4 (16)
B3—B4—B9—B8	−38.8 (11)	P2—C3—C8—C7	174.5 (10)
B5—B4—B9—B8	100.6 (13)	C3—P2—C9—C10	−67.1 (11)
C1—B4—B9—B10	−63.2 (14)	C2—P2—C9—C10	179.4 (10)
C2—B4—B9—B10	4.5 (16)	Hg1—P2—C9—C10	63.7 (10)
B3—B4—B9—B10	−99.6 (14)	C3—P2—C9—C14	173.0 (9)
B5—B4—B9—B10	39.8 (12)	C2—P2—C9—C14	59.5 (11)
C1—B4—B9—B3	36.4 (10)	Hg1—P2—C9—C14	−56.3 (10)
C2—B4—B9—B3	104.0 (12)	C14—C9—C10—C11	−54.7 (19)
B5—B4—B9—B3	139.4 (13)	P2—C9—C10—C11	−176.6 (13)
B7—B8—B10—B6	−36.6 (12)	C9—C10—C11—C12	57 (2)
B9—B8—B10—B6	100.3 (13)	C10—C11—C12—C13	−58 (2)
B3—B8—B10—B6	62.7 (17)	C11—C12—C13—C14	60 (2)
B2—B8—B10—B6	−0.4 (18)	C12—C13—C14—C9	−58.1 (19)
B7—B8—B10—B9	−136.9 (12)	C10—C9—C14—C13	54.4 (16)
B3—B8—B10—B9	−37.6 (12)	P2—C9—C14—C13	170.9 (11)
B2—B8—B10—B9	−100.7 (14)	C21—P1—C15—C20	−100.2 (11)
B9—B8—B10—B7	136.9 (12)	C1—P1—C15—C20	141.2 (10)
B3—B8—B10—B7	99.3 (13)	Hg1—P1—C15—C20	27.8 (11)
B2—B8—B10—B7	36.2 (12)	C21—P1—C15—C16	24.7 (12)
B7—B8—B10—B5	−99.7 (13)	C1—P1—C15—C16	−93.9 (11)
B9—B8—B10—B5	37.2 (12)	Hg1—P1—C15—C16	152.8 (9)
B3—B8—B10—B5	−0.4 (17)	C20—C15—C16—C17	−53.1 (18)
B2—B8—B10—B5	−63.5 (17)	P1—C15—C16—C17	−178.0 (11)
C2—B6—B10—B8	−64.0 (16)	C15—C16—C17—C18	57.3 (18)
B7—B6—B10—B8	36.9 (13)	C16—C17—C18—C19	−60.0 (19)
B5—B6—B10—B8	−100.4 (14)	C17—C18—C19—C20	59 (2)
B1—B6—B10—B8	−1.8 (17)	C18—C19—C20—C15	−56 (2)
C2—B6—B10—B9	0.3 (16)	C16—C15—C20—C19	53.2 (18)
B7—B6—B10—B9	101.3 (14)	P1—C15—C20—C19	−179.8 (12)
B5—B6—B10—B9	−36.1 (12)	C15—P1—C21—C22	68.0 (10)
B1—B6—B10—B9	62.6 (16)	C1—P1—C21—C22	−177.2 (9)
C2—B6—B10—B7	−101.0 (12)	Hg1—P1—C21—C22	−60.0 (9)
B5—B6—B10—B7	−137.4 (12)	C15—P1—C21—C26	−170.9 (10)
B1—B6—B10—B7	−38.7 (11)	C1—P1—C21—C26	−56.0 (12)
C2—B6—B10—B5	36.4 (11)	Hg1—P1—C21—C26	61.2 (11)
B7—B6—B10—B5	137.4 (12)	C26—C21—C22—C23	57.5 (16)
B1—B6—B10—B5	98.6 (12)	P1—C21—C22—C23	−178.5 (10)
B5—B9—B10—B8	137.9 (13)	C21—C22—C23—C24	−56.6 (19)
B3—B9—B10—B8	36.7 (11)	C22—C23—C24—C25	56 (2)
B4—B9—B10—B8	98.6 (13)	C23—C24—C25—C26	−59 (2)
B5—B9—B10—B6	36.2 (11)	C24—C25—C26—C21	58.5 (19)
B8—B9—B10—B6	−101.7 (14)	C22—C21—C26—C25	−57.1 (16)
B3—B9—B10—B6	−65.0 (15)	P1—C21—C26—C25	−175.5 (10)
B4—B9—B10—B6	−3.0 (17)		

### Hydrogen-bond geometry (Å, º)

**Table:**

*D*—H···*A*	*D*—H	H···*A*	*D*···*A*	*D*—H···*A*
B1—H1···Cl3^i^	1.10	2.71	3.791 (16)	169

Symmetry code: (i) −*x*, *y*+1/2, −*z*+1.

## References

[bb1] Flack, H. D. (1983). *Acta Cryst.* A**39**, 876–881.

[bb2] Sheldrick, G. M. (1996). *SADABS* University of Göttingen, Germany.

[bb3] Sheldrick, G. M. (2008). *Acta Cryst.* A**64**, 112–122. 10.1107/S0108767307043930 18156677

[bb4] Siemens (1996). *SMART* and *SAINT* Siemens Analytical X-ray Instruments Inc., Madison, Wisconsin, USA.

[bb5] Su, F.-F., Dou, J.-M., Li, D.-C. & Wang, D.-Q. (2007). *Acta Cryst.* E**63**, o3335.

[bb6] Su, F., Guo, Q., Dou, J., Li, D. & Wang, D. (2008). *Acta Cryst.* E**64**, m134. 10.1107/S1600536807065130 PMC2915082 21200491

[bb7] Yang, L. G., Zhu, C. C., Zhang, D. P., Li, D. C., Wang, D. Q. & Dou, J. M. (2011). *Polyhedron*, **30**, 1469–1477.

[bb8] Zhang, D.-P., Dou, J.-M., Li, D.-C. & Wang, D.-Q. (2006). *Acta Cryst.* E**62**, o418–o419.

